# Interpretation Bias Toward the Positive Impacts of Digital Interventions in Health Care. Comment on “Value of the Electronic Medical Record for Hospital Care: Update From the Literature”

**DOI:** 10.2196/37208

**Published:** 2022-03-04

**Authors:** Erfan Shakibaei Bonakdeh

**Affiliations:** 1 Department of Management Monash Business School Monash University Melbourne, VIC Australia; 2 Pharmacy Department Alfred Health Melbourne, VIC Australia

**Keywords:** cost analysis, costs and cost analyses, economic advantage, electronic medical records, electronic records, health care, hospitals, computerized medical records system, quality of health care, secondary data

The review paper by Uslu and Stausberg [1] certainly sheds some light on the positive impacts of electronic medical records (EMRs) in the hospital context. The emergence of such papers, as fruitful as they could be for minimizing skepticism among hospital executives and managers and compelling them to embark on the digitization journey, could introduce hasty and immature uptake of the technology, if biased. As a researcher heavily focused on health care digitization, I can debate that the results presented in the format of a review in the said paper are not impartial.

As highlighted by the literature, the improvement of processes caused by EMRs, as valuable as it is, may not contribute to patient outcome criteria (ie, mortality rate), and this has been one of the lengthiest debates in the field of digital health [2]. Thus, the authors’ statement “the review also showed improvements in quality of care by all respective studies” struck me as a great surprise. Further examination of the paper has brought to light that this statement was overpowered by some flaws in the study.

The bias toward declaring positive results from studies that either lacked or presented statistically insignificant positive outcomes (as noted in the original paper) is the major downside of this review. For example, Uslu and Stausberg [1] noted a positive association between EMR adoption and efficiency in the study by Adler-Milstein et al [3]. Surprisingly, in the original study, the authors clearly declared no significant association with regard to efficiency.

Additionally, Uslu and Stausberg [1] did not draw a clear line between the types of quality criteria (safety, timeliness, process, and patient outcomes), which not only is confusing to readers but also does not demonstrate the magnitude of improvement in each dimension. As such, a criterion such as mortality rate that does not normally show a significant improvement would be overpowered by process outcomes, which often behave reversely.

The aim of the study to “summarize empirical studies about the value of electronic medical records (EMRs) for hospital care” does not justify the inclusion of a few studies [1]. For example, “Higher rates of adoption of key EHR functions among high-quality hospitals” was reported as the result of Elnahal et al [4]. The aim of the said study can show the association between the presence of high quality in targeted hospitals and the presence of IT (information technology). Thus, it is not clear if high quality was derived by the EMR or whether high-quality hospitals adopted EMRs to maintain their status as a high-quality hospital. As the authors noted, “high quality and EHR adoption may be linked”; however, this is no strong evidence on which review studies can rely. On the other hand, the exclusion of computerized physician order entry (CPOE) was not explained by the authors as many EMRs already incorporate CPOE functions. By contrast, some studies included in the review, for example, Elnahal et al [4], mentioned the existence of CPOE in most high-quality hospitals in their sample.

To conclude, since the outcomes of secondary studies are often consulted by managers and politicians in the health care sector, researchers must be vigilant of the extensive impacts of research bias on fundamental decisions that they may cause.

## Abbreviations

CPOE: computerized physician order entry
EMR: electronic medical record
IT: information technology
